# Adaptive short‐term associative conditioning in the pancreatic β‐cell

**DOI:** 10.14814/phy2.14403

**Published:** 2020-03-31

**Authors:** Juan V. Sanchez‐Andres, Raquel Pomares, Willy J. Malaisse

**Affiliations:** ^1^ Department of Medicine Universitat Jaume I Castellon Spain; ^2^ Department of Physiology Universidad Miguel Hernandez Alicante Spain; ^3^ Department of Biochemistry Université Libre de Bruxelles Brussels Belgium

**Keywords:** carbamoylcholine, cholinergic, conditioning, insulin, memory, mouse pancreatic islet, β‐cells (beta‐cells)

## Abstract

This study associates cholinergic stimulation of the pancreatic β‐cell electrical activity with a short‐term memory phenomenon. Glucose pulses applied to a basal glucose concentration induce depolarizing waves which are used to estimate the evolution of the β‐cell glucose sensitivity. Exposure to carbamoylcholine (carbachol) increases the size of the glucose‐induced depolarizing waves. This change appears after carbachol withdrawal and implies a temporal potentiation of sensitivity (TPS) lasting up to one hour. TPS induction requires the simultaneous action of carbachol and glucose. The substitution of glucose with the secretagogues glyceraldehyde or 2‐ketoisocaproate mimics glucose‐induced TPS, while palmitate does not. TPS is not produced if the membrane is kept hyperpolarized by diazoxide. Glucose can be replaced by tolbutamide, suggesting a role of depolarization and a subsequent increase in intracellular calcium concentration. A role for kinases is suggested because staurosporine prevents TPS induction. Cycloheximide does not impair TPS induction, indicating that de novo protein synthesis is not required. The fact that the two inputs acting simultaneously produce an effect that lasts up to one hour without requiring de novo protein synthesis suggests that TPS constitutes a case of short‐term associative conditioning in non‐neural tissue. The convergence of basal glucose levels and muscarinic activation happens physiologically during the cephalic phase of digestion, in order to later absorb incoming fuels. Our data reveals that the role of the cephalic phase may be extended, increasing nutrient sensitivity during meals while remaining low between them.

## Graphical abstract

Cholinergic stimulation of the pancreatic β-cells electrical activity induces a short-term memory phenomenon that implies a temporal potentiation of sensitivity (TPS) lasting up to 1 hr. The requirement of two paired inputs (carbachol and glucose) allows suggesting that TPS constitutes a case of short-term associative conditioning in a nonneural tissue, and points to a generalization of associative mechanisms to nonneural systems. The convergence of basal glucose levels and muscarinic activation happens physiologically during the digestion cephalic phase. Our data allow hypothesizing that the role of the cephalic phase is extended, increasing nutrient sensitivity (system’s gain) after eating while being low between meals.

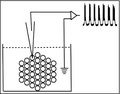

## 1 INTRODUCTION

It is generally accepted that a given cause induces an effect except in cases where the responsiveness of a system changes. Pavlov is believed to have provided the main historical milestone that challenged the cause–effect relationship when discovering associative memory. Pavlov showed that salivary secretion could be produced by a previously inactive stimulus (the sound of a ring), which implies that systems can respond differently. Pavlov's disciples (Planelles and Luwisch) sought parallel effects in other equally innervated systems (Planelles & Luwisch, 1935,1936). They suggested conditioned hypoglycemia as a parallel to classical Pavlovian experiments applied to the endocrine pancreas. The turbulence of the Spanish Civil War overshadowed the publication of this first description, thus it is frequently attributed to Mityushov (1954) (as cited by Overduin, Dworkin, & Jansen, 1997), who also demonstrated conditioned hypoglycemia following procedures that closely resembled Pavlov's. Likewise, classical conditioning experiments have shown that secretion of insulin can be controlled by stimuli in rats, dogs, and humans (Woods, Alexander, & Porte, 1972). Woods et al described conditioned hypoglycemia by inducing insulin secretion in rats in response to an olfactory stimulus (Woods, 1983).

Conditioned hypoglycemia is due to the secretion of insulin activated by the cholinergic fibers of the vagus nerve (Porte, Girardier, Seydoux, Kanazawa, & Posternak, 1973; Woods, 1972). Atropine or severing the vagi eliminates conditioning (Bergman & Miller, 1973; Woods, Hutton, & Makous, 1970). On the other hand, Cerasi (1981), Nesher and Cerasi (1987), Nesher, Eylon, Segal, and Cerasi (1989) demonstrated that the effect of β‐cell secretagogues is not limited solely to acute stimulation, coining the expressions time‐dependent inhibition (TDI) and time‐dependent potentiation (TDP) to describe the desensitization and sensitization that result in reduced or amplified insulin. These authors also found that these changes remained after withdrawing the agent. TDP has been well‐characterized (Nesher, Praiss, & Cerasi, 1988) and confirmed (Rasmussen et al., 1995). One case of TDP is called proemial sensitization, in which priming of insulin secretion was induced by acetylcholine applied at glucose concentrations incapable of producing TDP (Rasmussen et al., 1995; Zawalich, Zawalich, & Rasmussen, 1989). Thus, the existence of some memory in the islets of Langerhans (Zawalich, Diaz, & Zawalich, 1988; Zawalich & Zawalich, 1988), maybe responsible for the varying responsiveness that depends on the previous history of secretagogue exposure. Furthermore, this mechanism has been related to the pathophysiology of obesity (see ref. 18 for a review). The vagal ability to modulate insulin secretion has been confirmed (Gilon & Henquin, 2001), and there is consensus in considering vagal innervation as the part of the nervous system that stimulates ‘rest and digest’ and ‘feed and breed’ processes(Trajkovski & Wollheim, 2016) and is considered to be involved in the development of diet‐induced obesity (Lartigue, 2016).

While these advances concerning pancreatic responsiveness were made throughout the twentieth century, other notable advances contributed to our understanding of the vagal molecular effect. The pancreatic cholinergic innervation is part of the vagal branching (Woods & Porte, 1974). Cholinergic agents were first reported in 1967 to stimulate insulin release from pieces of rat pancreas incubated in the presence of a low concentration of glucose (5.6 mM). Thus, in the presence of glucose either acetylcholine or carbamoylcholine (carbachol) increased insulin output (Malaisse, Malaisse Lagae, Wright, & Ashmore, 1967). In this aforementioned study, atropine eliminated the insulin secretory response to the cholinergic agents, while not affecting that of glucose. Stimulation of insulin secretion by the cholinergic pathway involves a sophisticated sequence of cellular events, starting with the occupation of muscarinic receptors, followed by the activation of phospholipase C and the liberation of diacylglycerol and inositol 1,4,5‐triphosphate from membrane phosphoinositides, the activation of protein kinase C by diacylglycerol, and the mobilization of intracellular calcium ions by inositol 1,4,5‐triphosphate. These biochemical events lead to a remodeling of ionic fluxes and induction of bioelectrical activity (Malaisse, 1986). Later, it was reported that the administration of acetylcholine to the islets exposed to glucose changed in a rapid, sustained, and rapidly reversible manner, the bioelectric bursting pattern into one of continuous activity, the response to the cholinergic agent being opposed by atropine (Gagerman, Idahl, Meissner, & Täljedal, 1978). Likewise, in 1988, cholinergic agonist carbachol was found to stimulate the release of insulin, and cause, in the pancreatic β‐cells, an initial rapid depolarization with an increased rate of firing. Atropine suppresses the effects of carbachol on electrical activity (Sanchez‐Andres, Ripoll, & Soria, 1988).

Although the original descriptions of modifiability in the physiology of the endocrine pancreas are about 100 years old, three areas require further study: a. the existence of electrophysiological counterparts to these phenomena, b. the extent to which memory in the islets of Langerhans is an observable property at the cellular level (β‐cells), and c. the electrophysiological characterization at the cellular level of this effect. This research aims to provide insight into these unresolved questions and demonstrate that pancreatic β‐cells are a potential model of non‐neural associative conditioning. In this paper, we show that the application of carbachol (mimicking a vagal discharge) to isolated islets of Langerhans can modify their response to glucose. We suggest that pancreatic β‐cells are endowed with a modality of associative conditioning (Bliss & Collingridge, 1993), which shows some degree of parallelism with others described in neural systems (Bliss & Collingridge, 1993; Bliss & Lomo, 1973). This mechanism can play an essential physiological role, increasing the sensitivity of β‐cells during meals.

## 2 MATERIAL AND METHODS

### 2.1 Study approval

The experiments were carried out according to institutional animal care guidelines. Animal housing and all protocols were approved and in accordance with institutional guidelines, Spanish animal protection laws, and conformed to the Directive 2010/63 EU of the European Parliament. The experimental protocol was approved by the Ethics Committee for Research and Animal Welfare of the University Jaume I (License reference: 2015/VSC/PEA/00053). Mice were housed in standard conditions.

### 2.2 Experimental animals and islet preparation

OF‐1 albino, 2–4‐month‐old mice were used. The animals were killed by cervical dislocation. The islets of Langerhans were obtained as previously described (Andreu, Soria, & Sanchez‐Andres, 1997; Sanchez‐Andres, Malaisse, & Kojima, 2019). A complete laparotomy was done to extract the pancreas and then attach it to the bottom of a Petri dish. The islets were microdissected by hand with the help of a stereomicroscope. The Petri dish was filled with a modified Krebs solution with the following composition (in mM): 120 NaCl, 25 NaHCO_3_, 5 KCl, 2.6 CaCl_2_ and 1 MgCl_2_, and was equilibrated with a gas mixture containing 95% O_2_ and 5% CO_2_ at RT. In these conditions, viable islets could be obtained for more than 8 hr. The islets were transferred to the recording chamber after the microdissection.

### 2.3 Electrophysiological recording

The intracellular electrical activity (membrane potential) of β‐cells was recorded from the microdissected islets continuously perifused with the modified Krebs solution at 36ºC (pH 7.4). The volume of the chamber was 50 μl and the flow 1 ml/min, allowed for a fast washout of the applied drugs.

Borosilicate sharp microelectrodes (OD, 2.0 mM; ID. 1.0 mm, Sutter Instruments, Co.) were pulled with a Narishige PE2 puller (Narishige, Japan). The electrodes were filled with 3 M potassium citrate and 50 mM KCl. An Axoprobe microelectrode amplifier (Axon Instruments) was used to perform electrophysiological recordings. Data were acquired at 1 kHz frequency sampling using Clampex software (v10.6, Molecular Devices) through an acquisition card (Digidata 1,550, Molecular Devices), and stored on computer hard disk for further analysis using ClampFit (v10.6, Molecular Devices) and/or Origin Lab (Origin Pro 2018).

**Figure 1:**
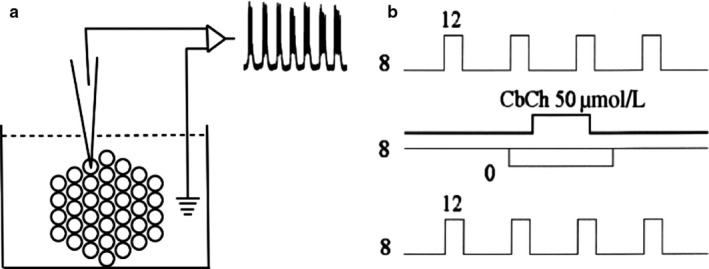
(a) Experimental system. Microdissected islets of Langerhans are attached with micropins to the bottom of the recording chamber. The sharp microelectrode accesses the chamber from the top. After cell impalement, the signal is led to the amplifier that subtracts the signal coming from the bath (ground). The resulting signal is seen in an oscilloscope and stored in a hard disk. (b) Protocol diagram for examining changes in temporal glucose sensitivity. *Top illustration*. On a basal 8 mM glucose concentration, one‐minute long, 12 mM glucose pulses are applied; each of them separated from the others by 5 min. *Middle illustration*. Experimental manipulation. On the basal 8 mM glucose concentration, carbachol is applied for 5 min and waiting 5 min to allow washout. *Bottom illustration*. Same as in the *top illustration* the response of 1‐min 8 to 12 mM glucose pulses is obtained to check whether the manipulation as in the *middle ilustration* has produced a change in responsiveness. Note that time scales are not realistic

Islet cells were impaled in the presence of 10 mM glucose in a modified Krebs solution to identify cells exhibiting robust oscillatory electrical behavior typical of β‐cells (Figure 1a). β‐cells were further identified by the characteristic membrane hyperpolarization and cessation of electrical activity upon perfusion without glucose. Data from 89 recorded cells were included in the study.

Given the requirement of long‐lasting experiments, strict criteria for considering a recorded cell as acceptable were applied: a cell input resistance higher than 100 MΩ, and membrane potential of less than 50 mV, stable for at least 15 min before starting the experimental protocols.

### 2.4 Statistical analysis

Short‐lasting glucose pulses (1‐min long, 8 to 12 mM) (Figure 1b) produced transient wave‐like depolarizations. Areas under the wave‐like glucose‐induced depolarizations (AUD) were measured. Specific protocols are detailed in the first part of the results section, where the setting of a procedure to analyze sensitivity to glucose is described. Unpaired *t*‐tests were performed to compare the AUD differences statistically before and after the experimental manipulations. A critical significance level α of 0.05 was selected before data analysis (Table 1). Statistical analysis was done with Prism v 7 (GraphPad Software, Inc.).

**Table 1:** Mean and *SD* of the experimental conditions where TPS was observed

	CbCh 50 µM (*n* = 9)	Glyceraldehyde 10 mM (*n* = 4)	KIC 10 mM (*n* = 8)	Leucine (10 mM) (*n* = 4)	Tolbutamide (50 μM) (*n* = 4)	Cycloheximide (50 μM) (*n* = 3)
Controls	0.98 (*SD* 0.20)	1.00 (*SD* 0.13)	1.00 (*SD* 0.13)	0.99 (*SD* 0.12)	0.99 (*SD* 0.18)	0.97 (*SD* 0.07)
1st pulse (5.5 min)	1.62 (*SD* 0.42)*	1.87 (*SD* 0.04)*	1.86 (*SD* 0.12)*	2.03 (*SD* 0.21) ns	1.39 (*SD* 0.20)*	1.56 (*SD* 0.29)*
2nd pulse (11.5 min)	1.56 (*SD* 0.29)*	1.75 (*SD* 0.04)*	1.35 (*SD* 0.39) ns	1.36 (*SD* 0.07) ns	1.27 (*SD* 0.11)*	1.38 (*SD* 0.20)*
3rd pulse (17.5 min)	1.35 (*SD* 0.21)*	1.33 (*SD* 0.21) ns	1.19 (*SD* 0.33) ns	1.27 (*SD* 0.11) ns	1.22 (*SD* 0.08) ns	1.28 (*SD* 0.16)*
4th pulse (23.5 min)	1.39 (*SD* 0.28)*	1.49 (*SD* 0.03)*	1.07 (*SD* 0.27) ns	—	1.31 (*SD* 0.27) ns	1.15 (*SD* 0.17) ns
5th pulse (29.5 min)	1.31 (*SD* 0.20)*	1.27 (*SD* 0.25) ns	1.24 (*SD* 0.04)*	—	1.21 (*SD* 0.22) ns	1.27 (*SD* 0.11)*

Note

The experiments were carried out as described in Methods. Several (3 to 5) control pulses consisting of one‐minute long steps to 12 mM glucose from a basal concentration of 8 mM glucose were applied. 5 min was the time allowed to pass between pulses. Then, all the experimental conditions described in Results were performed, and, later, again, one‐minute glucose steps from 8 to 12 mM glucose, separated by 5 min, were applied. These steps produced transient depolarizations in which the area was measured (AUD). To obtain control values, the measured areas for every experimental condition were normalized over one and then pooled and averaged. Likewise, the AUDs obtained from the experimental conditions were normalized over one, pooled and averaged. The table shows the results obtained for 5 consecutive glucose pulses applied after removing the TPS‐inducing agent. Timing is, thus, 5.5, 11.5, 17.5, 23.5, and 29.5 min because i.e., in the case of the first pulse, 5 min were allowed to pass after removing the drug and/or returning to basal 8 mM glucose. Then, this pulse was applied at minute 5 and lasted for 1 min. To this end, this pulse is labeled as 5.5 min ending at minute 6. 5 min were allowed to pass before applying the second pulse, at minute 11, and lasting for 1 min. Then, the second pulse is labeled as 11.5 min, and so on.

*the *p*‐value is less than 0.05 indicating a statistically significant difference; ns, the p‐value is greater than 0.05 indicating a no statistically significant difference.

All the reagents were obtained from Sigma.

## 3 RESULTS

### 3.1 Setting up a procedure to test dynamic changes in the bioelectric sensitivity of pancreatic β‐cells to glucose

**Figure 2:**
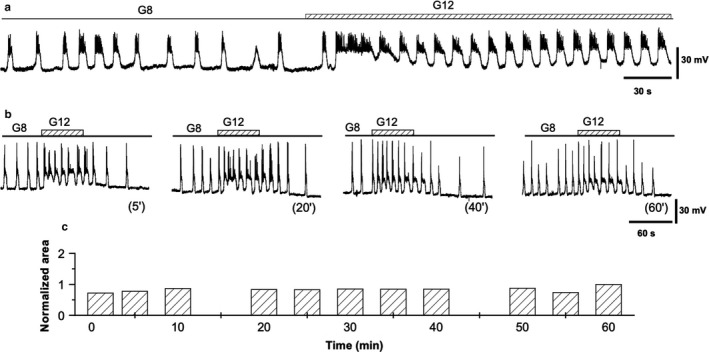
Pancreatic β‐cell responsiveness to steady glucose concentrations and glucose steps. (a) A typical response to sustained glucose concentrations. 8 mM glucose causes a pattern of alternating silent (hyperpolarized) and active (depolarized) phases. An increase in 12 mM glucose extends the active phases at the expense of the silent ones. (b) Four responses to 1‐min steps from 8 to 12 mM glucose concentrations. Striped horizontal bars indicate the 1‐min glucose increase from 8 to 12 mM. The time of the applied pulse is indicated in parentheses. (c) Reproducibility of the responsiveness to glucose steps expressed in terms of the area under depolarization (AUD) over 1 hr. The time scale corresponds to that shown in b. Thus, bars at 5, 20, 40, and 60 min are the AUD measured in the pulses as labeled in b. All the traces in the figure were obtained from the same β‐cell. Note that time scales in a and b are different

At glucose concentrations ranging from 7 to 20 mM, oscillatory activity of pancreatic β‐cells typically consist of hyperpolarized (silent) phases and depolarized (active) phases crowned by calcium action potentials both in vitro (Ashcroft & Rorsman, 1989) and in vivo (Sanchez‐Andres, Gomis, & Valdeolmillos, 1995). Increases in the concentration of glucose prolong the active phases at the expense of the silent ones (Figure 2a). Transient glucose pulses (minutes) result in depolarizing waves that last somewhat longer than the pulse duration (Figure 2b). These waves are associated with a transient increase in activity (longer active phases) that last until the return to membrane potential baseline levels. Good quality records (which meet the quality criteria specified in Methods) allowed us to consistently record stable depolarizing waves for up to two hours (Figure 2b and 2c).

**Figure 3:**
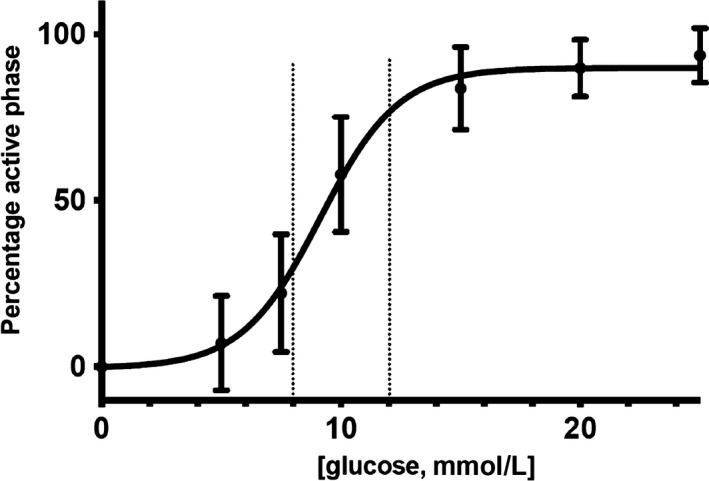
Dose–response for glucose, measuring the percentage of time in the active phase. 8 and 12 mM glucose concentrations are indicated with dotted lines

The gold standard to estimate the sensitivity of a given system to an agent is to obtain dose–response curves where increasing concentrations of the agent lead to corresponding responses. These curves can often be fitted nonlinearly, allowing for parameters like the Hill slope, the IC50 and the saturation level to be obtained (Figure 3). For the electrical response of the pancreatic β‐cell, this approach has been successfully used, measuring the percentage of time in the active phase (%AP) as a function of the glucose concentration (Atwater, Carroll, Xu, & Li, 1989; Sanchez‐Andres et al., 1995). Despite the optimality of this procedure to check glucose sensitivity of pancreatic β‐cells, it is not useful when testing transient changes in sensitivity. Electrophysiological records can hardly be stable for more than 1 to 2 hr. Then, characterizing changes in glucose sensitivity in this time frame is limited because building dose–response curves requires testing different glucose concentrations and waiting for stabilization of each of them. Given the slow pace of these processes (minutes), a change in sensitivity in this time frame (and/or around 1 hr) will contaminate the data. One alternative is to test the evolution over time of the responses to identical stimuli assuming the limitations of observing just one region in the curve. The challenge is, then, to select the most representative region in the dose–response curve. Preliminary experiments were performed (data not shown) to check different conditions of the β‐cell electrical response to glucose concentrations in steps. The findings indicate that jumps from 8 to 12 mM glucose over 1 min caused a reproducible change in the electrical activity (as in Figure 2b and Figure 2c) and these are used as controls for sensitivity changes induced by experimental agents. This change takes the form of a depolarizing wave that can be measured (area under the depolarization, AUD). The rationale for this approach relies on the following three assumptions: a. the range 8–12 mM glucose falls in the region of the steeper slope on the dose–response curve (Figure 3); b. these glucose concentrations make physiological sense because they are possible in the normal run of the β‐cells. Frequently, dose–response curves are built from no glucose to 20 or more mM glucose concentrations. Indeed, this range allows to complete the curves but far from the physiological range as euglycemia is strictly controlled and is not usually lower than 6 mM (Sanchez‐Andres et al., 1995) except in hypoglycemic conditions associated with pathology; c. 8 to 12 mM steps imply exposure to relatively high glucose concentrations that do not reach (Figure 3) the saturation level. Thus, this step change from 8 to 12 mM glucose during a short exposure period can be a useful indicator of changes in pancreatic β‐cell glucose sensitivity. Furthermore, such a short‐lasting experimental manipulation can be done repeatedly together with the expected duration of stable intracellular impalements. This approach involves applying sequentially 8 to 12 mM glucose steps quantifying the effect by measuring the AUD, and waiting long enough to allow the cell to return to control conditions. We estimated, from preliminary experiments, the optimal duration of the steps to be 1 min and the time between steps, 5 min, in order to wait for the system to return to baseline. A protocol such as that described in Figure 1b may be valid, provided the internal cell variability is smaller than that expected from sensitivity‐induced changes.

To characterize internal system variability, we applied 3 to 5 1‐min‐long pulses, separating one from the other by 5 min to seven cells (*n* = 36 pulses), and after normalization over 1, we obtained a standard deviation average of 0.18; lower 95% CI of mean: 0.93; upper 95% CI of mean: 1.05. Changes in the measured area which are greater than 20% of controls will derive from the experimental treatment, not from the β‐cell intrinsic variability.

### 3.2 Modifiability of the glucose responsiveness by carbachol application

**Figure 4:**
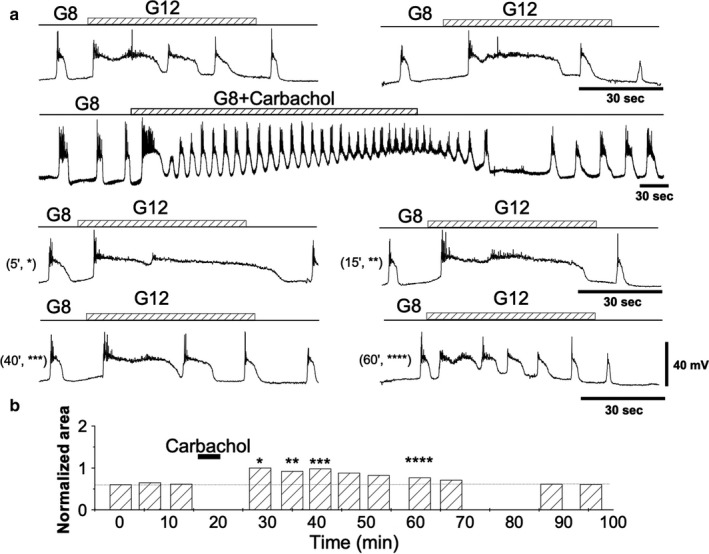
Modifiability of glucose responsiveness by carbachol. (a)* First row*. Two representative control traces obtained by applying 1‐min 12 mM glucose pulses on an 8 mM glucose. *Second row*. A quantity of 50 μM carbachol is applied for 5 min on 8 mM glucose concentration. *Third and fourth rows*. After carbachol washout, 1 min, 12 mM glucose pulses were applied as in the first row. The time after carbachol withdrawal is indicated in parenthesis. (b) Areas under the depolarizations (AUD) measured before and after carbachol application. Bars labeled with asterisks correspond to the identically labeled records in A (third and fourth rows). All the traces in the figure were obtained from the same β‐cell

**Figure 5:**
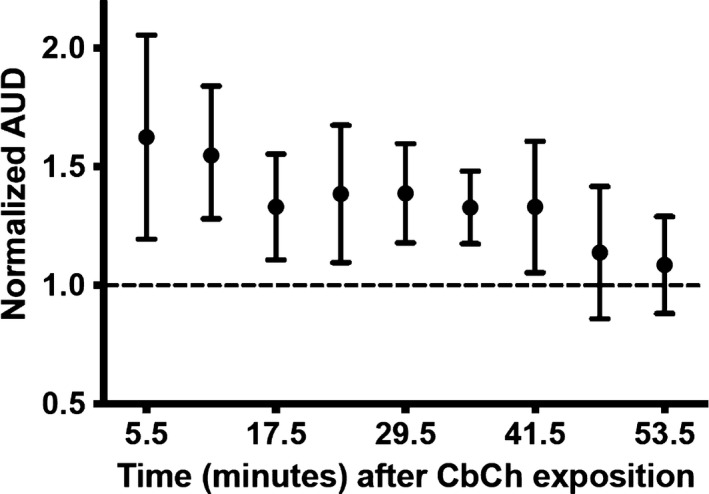
Temporal evolution of TPS. Mean and *SD* of the areas under depolarization (AUD) (*n* = 9) after carbachol application. The areas obtained for control pulses were normalized to 1 (broken line) to be used as a reference to calculate the normalized values for the pulses applied after carbachol. At least three control pulses, usually 4–5, were applied before carbachol administration

1‐min long pulses from a glucose concentration of 8 to 12 mM induced wave‐like depolarizations (Figure 4a, *first row*; Figure 4b, *first three bars*). A quantity of 50 μM carbachol applied for 5 min produced a rapid, sustained, and rapidly reversible increase in spiking activity to islets exposed throughout the experiment to 8.0 mM D‐glucose (Figure 4a, *second row*). The test pulses began 5 min after removing the carbachol to allow for a complete washing of the neurotransmitter, which was confirmed by the return of the membrane potential to the baseline. The response to these pulses progressively declined toward control levels in 40–60 min (Figure 4a, *bottom rows*, and Figure 4b). The cumulative responses from different experiments (*n* = 9) are shown in Figure 5 and Table 1. This result indicates that carbachol induces a lasting increase in the glucose sensitivity of the β‐cells (Temporal Potentiation of Sensitivity, TPS). TPS tends to return gradually to baseline levels in 30–60 min.

**Figure 6:**
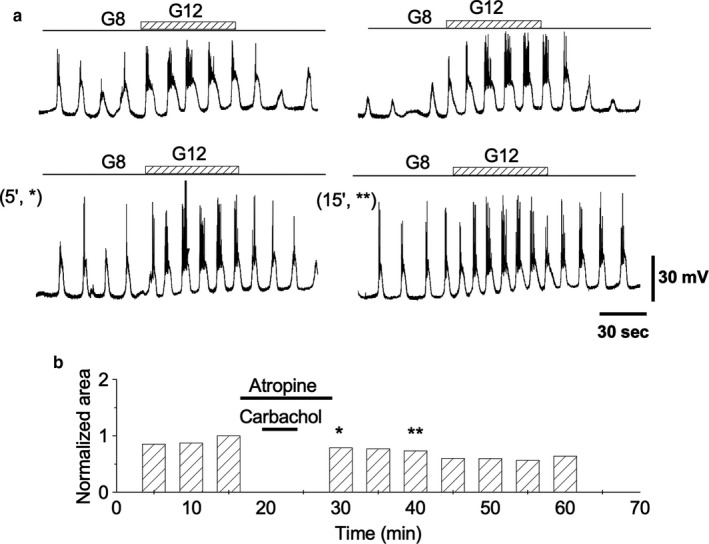
Atropine prevents TPS establishment. (a) Wavelike‐induced depolarizations by 8 to 12 mM glucose steps before (*top row*) and after (*bottom row*) carbachol treatment in the presence of atropine (10 μM). (b) Analysis of the areas under depolarization (AUD) when carbachol was applied together with atropine (see text for details on timing for atropine application). Bars labeled with asterisks correspond to the similarly labeled records in (a), *bottom row*. All the traces in the figure were obtained from the same β‐cell

The specificity of the carbachol action through muscarinic receptors was checked by applying carbachol in the presence of 10 μM atropine. Figure 6 shows that when the protocol was carried out in the presence of atropine, carbachol was unable to induce any increase in β‐cell responsiveness to glucose pulses (*n* = 10).

### 3.3 Glucose requirement for TPS induction

**Figure 7:**
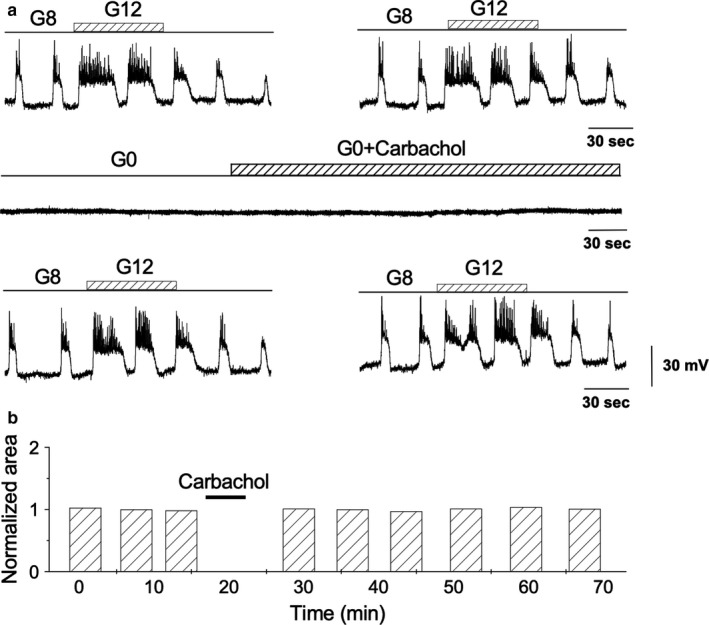
Effect of carbachol application in the absence of glucose. (a)* Top row*. Two representative traces of 1‐min long, 12 mM glucose pulses applied on 8 mM glucose.* Middle row*. Glucose is removed from the perfusing solution (see also Figure 3) and, then, 50 μM carbachol is applied for 5 min. Note the absence of carbachol effect.* Bottom row*. After carbachol washout, 8 mM glucose concentration is added to the perifusion solution, and 1‐min long, 12 mM glucose pulses were applied as in the top row. (b) Comparison between the areas under the depolarizations (AUD) induced by glucose pulses before and after carbachol treatment in the absence of glucose. All the traces in the figure were obtained from the same β‐cell

The protocols typically used to induce associative conditioning require the temporal convergence of two inputs in a given system (Alkon et al., 1992). The protocol used for TPS induction involved the simultaneous and transitory effects of two secretagogues: glucose and carbachol. The question arises as to whether TPS results only from carbachol action or if it requires the joint action of both inputs. The answer to this question was obtained by removing the glucose from the perifusion solution before applying carbachol (Figure 7a). After removing carbachol, basal glucose (8mM) was restored, and glucose pulses (12 mM) were applied to compare with the responses obtained before carbachol application (Figure 7a, *top row *versus* bottom row*). Carbachol applied in the absence of glucose was unable to produce TPS (Figure 7a and Figure 7b) (*n* = 10). Consequently, TPS induction requires the presence of carbachol and glucose simultaneously.

### 3.4 TPS induction by other nutrient secretagogues

**Figure 8:**
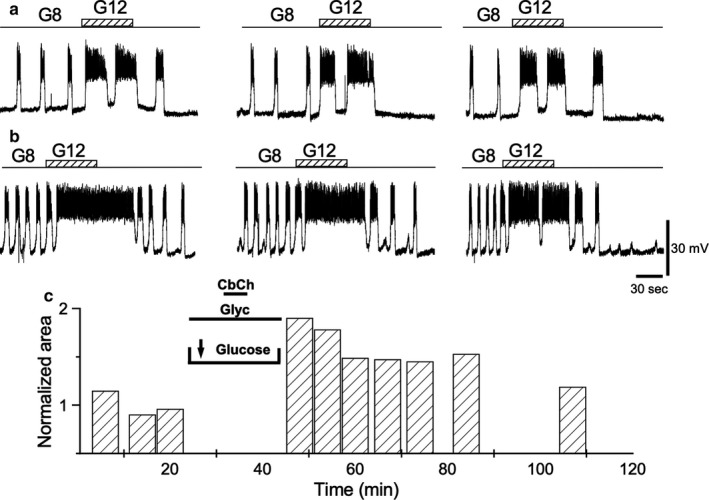
Effect of glyceraldehyde (Gly). (a) Control representative depolarizations induced by 1‐min long steps from 8 to 12 mM glucose. After control pulses, glucose is replaced by 10 mM glyceraldehyde before administering 50 μM carbachol, which is maintained for 5 min. (b) Steps as in (a) after 5 min of replenishing the 8 mM glucose and removing the glyceraldehyde. (c) AUD measurements throughout the experiment. All traces in the figure were obtained from the same β‐cell

**Figure 9:**
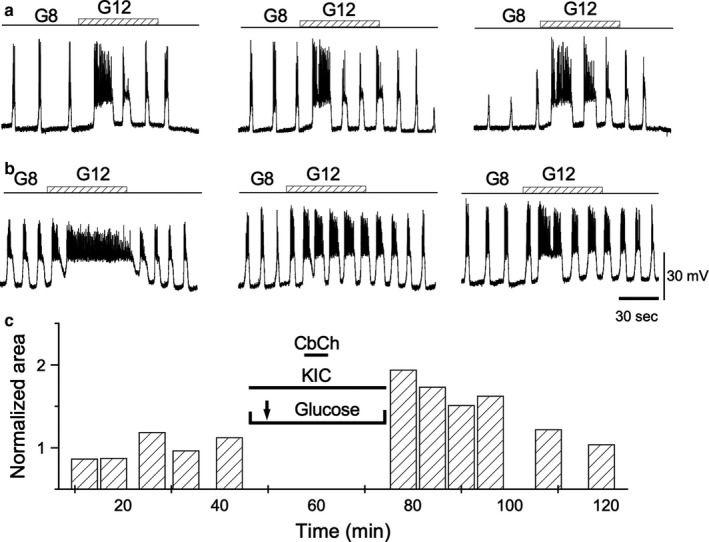
Effect of ketoisocaproate (KIC). (a) Representative control depolarizations induced by 1‐min long steps from 8 to 12 mM glucose. After control pulses, glucose is replaced by 10 mM KIC before administering 50 μM carbachol, which is maintained for 5 min. (b) Steps as in (a) after 5 min of replenishing the 8 mM glucose and removing the KIC. (c) AUD measurements throughout the experiment. All traces in the figure were obtained from the same β cell

**Figure 10:**
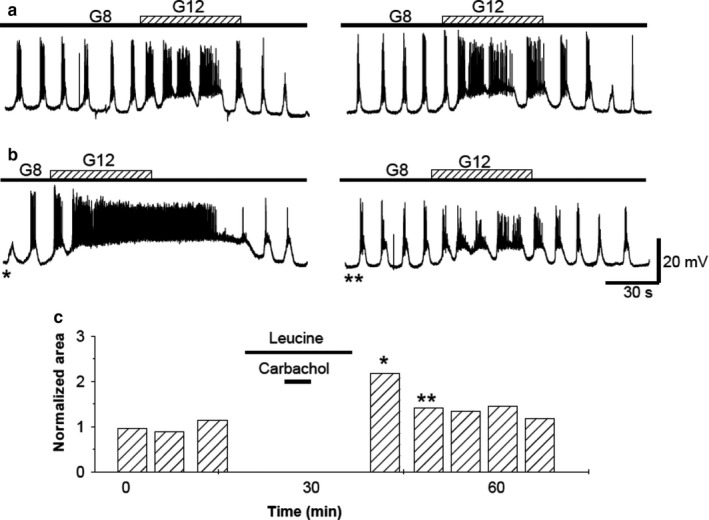
Effect of leucine. Leucine does not consistently produce TPS. The figure shows a representative case where TPS is induced. (a) 1‐min glucose control steps 8 to 12 mM. After the control pulses, glucose is replaced by 10 mM leucine before administering 50 μM carbachol, which is maintained for 5 min. (b) Steps as in A after 5 min of replenishing the 8 mM glucose and removing the leucine. (c) AUD measurements throughout the experiment. Bars labeled with asterisks correspond to the identically labeled records in (b). All traces in the figure were obtained from the same β‐cell

When carbachol was applied in the absence of glucose, but in the presence of either 10 mM glyceraldehyde (Figure 8) (*n* = 4) or 10 mM 2‐ketoisocaproate (Figure 9) (*n* = 8), a subsequent intensification of the bioelectrical response to a rise in D‐glucose concentration was observed. The cumulative responses for both secretagogues are shown in Table 1. The substitution of 8.0 mM D‐glucose for 10 mM l‐leucine at the time of carbachol administration did not intensify the bioelectrical response to the rise in glucose concentration from 8.0 to 12.0 mM consistently. The experiment was replicated in four cells, and the potentiation was observed only consistently in two of them (Figure 10). Table 1 provides the values for the test pulses under the leucine effect when TPS was induced. These data highlight a tendency that was not confirmed (see Discussion). In the other two cells, the potentiation could not be observed (data not shown; see Discussion). Exposing the islets to palmitate (625 μM) during carbachol administration did not produce any significant increase in response to test pulses (*n* = 6; data not shown).

### 3.5 TPS induction dependence on membrane potential

**Figure 11:**
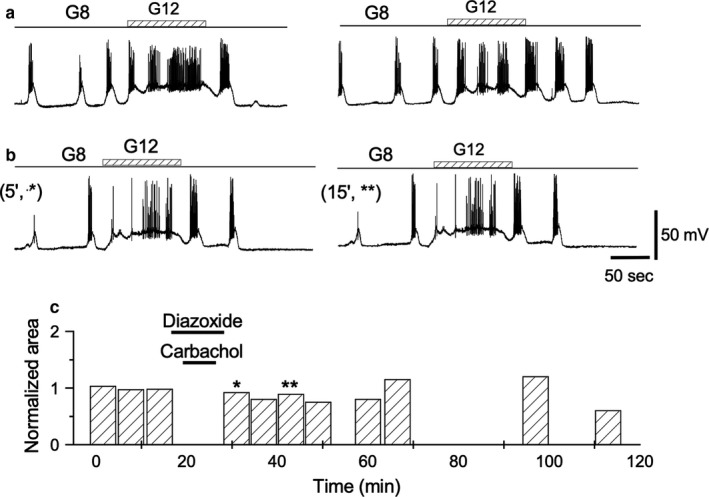
Effect of diazoxide. (a) 1‐min long glucose steps 8 to 12 mM before (top row) and after (bottom row) carbachol treatment in the presence of diazoxide (20 μM). (b) Analysis of the areas under the wavelike‐induced depolarizations (AUD) when carbachol was applied in the presence of diazoxide. Bars labeled with asterisks correspond to the similarly labeled records in (b). All the traces in the figure were obtained from the same β‐cell

The best‐known case of cellular associative conditioning is probably long‐term potentiation (LTP) (Bliss & Collingridge, 1993; Bliss & Lomo, 1973), extensively studied in neural systems. The LTP induction implies that two stimulus act at the same time on one cell. It is known that the role of one of the two associated inputs provides an appropriate level of depolarization while the other acts. This role has been experimentally tested, replacing one of the inputs by a depolarization, induced either pharmacologically or through depolarizing current injection. The latter technique is difficult when studying the pancreatic β‐cell because the sharp electrodes used for impalements cannot reliably drive the membrane potential towards a steady depolarized level. However, diazoxide and tolbutamide are known to change the membrane potential by acting on K‐ATP channels. The K‐ATP channels are the decisive step in glucose metabolism. Without glucose this channel stays open, and the cells hyperpolarize. Conversely, glucose metabolism increases the ATP/ADP ratio closing the channels and inducing cell depolarization and insulin release (Wu, Ding, Wang, & Chen, 2020). Diazoxide keeps the channels open even in the presence of glucose, forcing the cells to stay hyperpolarized. Tolbutamide works in the opposite way, closing the channels and depolarizing the cells even without glucose. Consequently, the use of these drugs acting distally on the membrane may be critical to test the need for intermediate stages of glucose metabolism in the induction of TPS. To verify the effect of maintaining the membrane hyperpolarized during carbachol administration, the following protocol was used: Several (3 to 5) control glucose pulses were applied before adding 20 μM diazoxide to the superfusion solution (including 8 mM glucose) and waiting 3 to 5 min until the diazoxide‐induced hyperpolarization was established. Then, 50 μM carbachol was applied for 5 min. Both carbachol and diazoxide were removed from the superfusion solution. Recovery of diazoxide hyperpolarization was usually done in the range of 5–7 min. Then, 8 to 12 mM glucose steps were applied and compared with controls. The absence of TPS due to diazoxide suggests (Figure 11) that depolarization of the plasma membrane was necessary for TPS to occur (*n* = 5).

**Figure 12:**
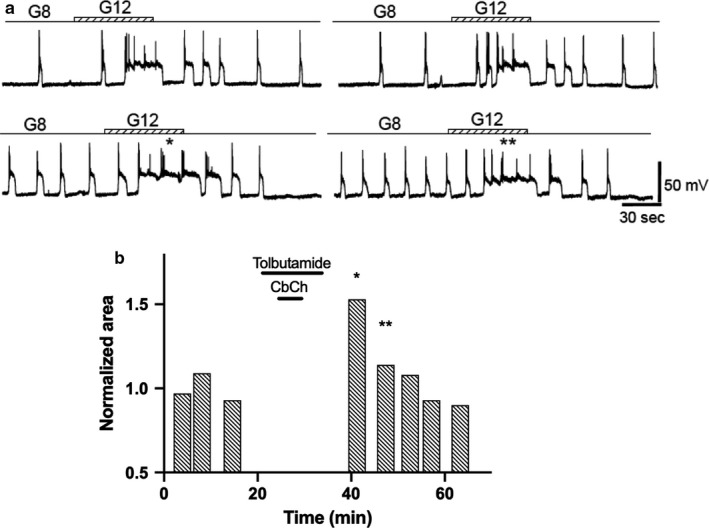
Effect of tolbutamide. Effect of carbachol application in the absence of glucose, but in the presence of 50 μM tolbutamide. (a)* Top row*. 1‐min glucose control steps 8 to 12 mM. After control pulses, glucose is replaced by 50 μM tolbutamide. When the depolarizing effect of tolbutamide is established, 50 μM carbachol is added for 5 min. Then, tolbutamide is removed, and an 8 mM glucose concentration is added to the perifusion solution.* Bottom row*. Steps as in the *top row*, after 5 min of replenishing the 8 mM glucose and removing the tolbutamide. (b) AUD measurements throughout the experiment. Bars labeled with asterisks correspond to the similarly labeled records in A,* bottom row.* All the traces in the figure were obtained from the same β‐cell

A mirror image of that recorded in the β‐cells exposed to diazoxide was observed when the cholinergic agent was administered to β‐cells exposed to 50 μM tolbutamide (Figure 12) (*n* = 4) without glucose. Thus, in these cases, a later potentiation of glucose‐induced bioelectrical activity was seen. The cumulative responses in the presence of diazoxide and tolbutamide are shown in Table 1. The potentiation in the glucose response after tolbutamide and carbachol removal indicates that the critical requirement to be associated to the cholinergic agonist is the depolarization or its intracellular consequences, rather than the mechanisms secondary to glucose metabolism. TPS induced in the presence of tolbutamide exhibits similar properties to that induced by glucose, with maximal values obtained for the first stimulus after carbachol withdrawal and a progressive decline toward the baseline over a 30–60‐min period.

The temporal potentiation of β‐cell sensitivity to glucose may be a Ca^2+^‐sensitive process modifying the Ca^2+^‐dependence of the β‐cell electrical activity (Ribalet & Beigelman, 1981; Sanchez‐Andres et al., 1988). Indeed, it was not observed when Ca^2+^ entry during carbachol application was blocked by 5 mM Co^2+^ (*n* = 4; data not shown). This requirement seems to be precisely on the extracellular calcium entry as 1 μM caffeine, capable of increasing the intracellular Ca^2+^ concentrations by releasing the cation from intracellular stores, could not produce TPS (*n* = 5; data not shown).

### 3.6 Blocking TPS induction by staurosporine

**Figure 13:**
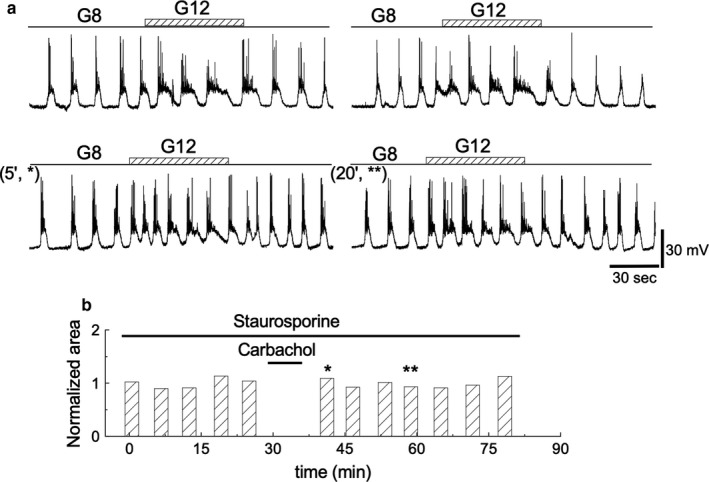
Effect of staurosporine. (a) 1‐min glucose steps of 8 to 12 mM before (upper row) and after (lower row) carbachol treatment in the presence of 30 nM staurosporine throughout the experiment. (b) AUD measurements throughout the experiment. The bars labeled with asterisks correspond to the records labeled in the same way in A, *bottom row*. All traces in the figure were obtained from the same β‐cell

TPS implies a sustained effect that lasts beyond the application of carbachol. This finding suggests the participation of some intracellular molecules capable of remaining activated after carbachol withdrawal. Several kinases are thought to be responsible for this type of sustained responses (Rasmussen et al., 1995; Wang & Feng, 1992). To verify the participation of kinases in the induction of TPS, we performed experiments with 30 nM staurosporine. Under these conditions, TPS was not induced (Figure 13) (*n* = 3). This result suggests that kinases are involved in TPS induction. One of them, the PKC, has been shown to produce sustained effects. To test this possibility, we conducted experiments (*n* = 7) using the phorbol ester 2 μM PMA. Regardless of the incubation time (up to 1 hr), the induction of TPS was not seen.

### 3.7 De novo protein synthesis is not required for TPS induction

**Figure 14:**
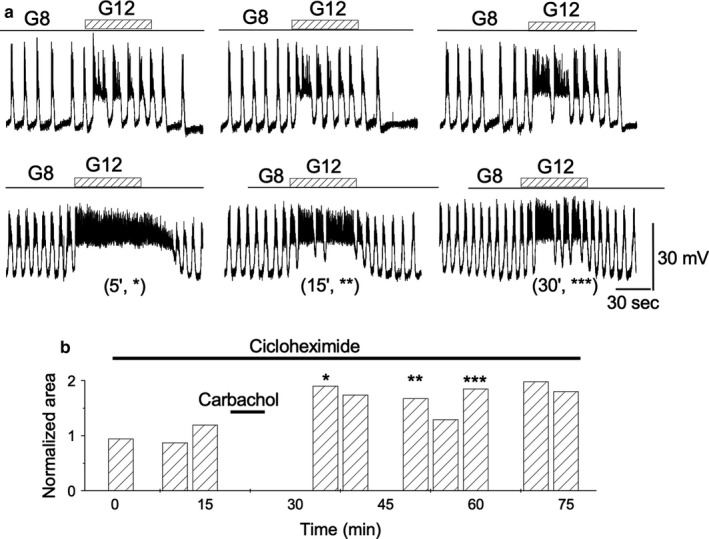
Effect of cycloheximide. (a) A quantity of 50 μM cycloheximide was added to the perfusion solution 2 hr before starting the protocol and maintained throughout the experiment. *Top row*. 1‐min glucose control steps 8 to 12 mM. *Bottom row*. After carbachol removal, glucose pulses as in the *top row* were applied. (b) AUD measurements throughout the experiment. The bars labeled with asterisks correspond to the records labeled in the same way in (a), bottom row. All the traces in the figure were obtained from the same β‐cell

It is generally accepted that long‐term changes in synaptic transmission require activation of de novo protein synthesis while short‐term changes do not. TPS duration indicates that it is a short‐term change. The execution of the experiments in the presence of the protein synthesis blocker cycloheximide allows for this possibility to be tested. The islets were preincubated in 50 μM cycloheximide for two hours before running the protocols, and the drug was kept in the perifusion medium throughout the experiment (*n* = 3). Cycloheximide was not able to prevent TPS induction (Figure 14), suggesting its independence from de novo protein synthesis. Table 1 provides the cumulative responses in the presence of cycloheximide.

## 4 DISCUSSION

The parasympathetic effect on the endocrine pancreas has been well characterized. Acetylcholine and its analogs are known to increase insulin release both in vitro and in vivo (Kajinuma, Kaneto, Kuzuya, & Nakao, 1968; Malaisse et al., 1967; Sharp, Culbert, Cook, Jennings, & Burr, 1974). Stimulation of the parasympathetic vagus nerve leads to an increase in insulin secretion (Bergman & Miller, 1973; Frohman, Ezdinli, & Javid, 1967; Kaneto, Kosaka, & Nakao, 1967; Porte et al., 1973) blockable by atropine. This increase is mediated through muscarinic receptors (Gilon, Nenquin, & Henquin, 1995; Malaisse, 1986).

The preabsortive or cephalic phase of insulin secretion is mediated by the muscarinic action of acetylcholine released by the vagus nerve (Rasmussen, Zawalich, Ganesan, Calle, & Zawalich, 1990). It can be assumed that this effect happens in the presence of a basal glucose concentration (around 6–8 mM) since it takes place before hyperglycemia is induced by nutrient absorption. We have mimicked vagal activation by applying the cholinergic agonist carbachol for 5 min together with 8 mM glucose and compared the 8 to 12 mM glucose‐induced depolarizing waves before and after carbachol treatment. The depolarizing waves were larger after the treatment and returned gradually to control levels after 30–‐60 min. This increase in the size of the depolarizing waves implies increased responsiveness of the system, as the stimuli were identical before and after carbachol treatment. The potentiation in glucose response is muscarinic because it is blocked with atropine. Applying carbachol without glucose did not change β‐cell responsiveness to glucose. All these data taken together show that the cell's response to glucose is not fixed but is temporally modified by the combined effect of glucose and carbachol, and neither can produce the effect when applied separately. The modification takes the form of a temporary potentiation of sensitivity (TPS) for less than one hour.

This temporary change in sensitivity can be interpreted as a short‐term memory mechanism. Such a temporary potentiation of sensitivity is compatible with the view that a vagal discharge occurring in the cephalic phase of digestion, when a meal is predicted to occur, causes β‐cells to increase their glucose response and optimize their responsiveness. In other words, the gain (sensitivity) in the system will be maximized at meals and will be minimal between meals. This mechanism will share neural properties with classical Pavlovian learning and can explain the early Planelles and Luwisch (1935, 1936) description that was later widely characterized by Woods as conditioned hypoglycemia (Woods, 1983, 2013).

The potentiation of glucose sensitivity induced by carbachol may also occur in the case of other secretagogues. Glucose induces insulin secretion by activating the so‐called canonical or initiating pathway (Henquin, 2000) for insulin secretion which blocks K‐ATP channels causing cell depolarization. The potentiation could be expected to be reproduced in cases where the pathways involved are similarly affected. Insulin secretion is stimulated by nutrients such as glucose, amino acids, and free fatty acids, as well as incretin hormones to adapt glucose homeostasis in response to a meal. While a thorough exploration of the possibilities is beyond the scope of this paper, we have tested several representative substrates. Glyceraldehyde, a lateral end‐product of glycolysis, mimics the effect of glucose on insulin secretion (Taniguchi, Okinaka, Tanigawa, & Miwa, 2000). Glyceraldehyde allows TPS induction by carbachol indicating that TPS does not require the steps associated with glycolysis to occur. α‐Ketoisocaproic acid (KIC) is a metabolite of leucine that induces insulin secretion from β‐cells (Panten, Kriegstein, Poser, Schönborn, & Hasselblatt, 1972). KIC directly inhibits the K‐ATP channels in pancreatic β‐cells, while it is still unclear whether direct inhibition of the K‐ATP channels by KIC contributes to insulin release (Heissig, Urban, Hastedt, Zünkler, & Panten, 2005). In our experiment, KIC allowed TPS induction while its precursor leucine did not do so consistently, failing in half of the cases. This ratio is reasonably adjusted to that described for leucine‐induced insulin secretion in single β‐cells (39.2%) (Hiriart, Sanchez‐Soto, Ramirez‐Medeles, & Malaisse, 1995). This indicates the existence of two β‐cell subpopulations based on their ability to respond to leucine. Research shows that one mechanism by which leucine induces insulin secretion is through its conversion to KIC (Newsholme, Brennan, & Bender, 2006). However, leucine and KIC also stimulate insulin release through different mechanisms (Gao et al., 2003). Recently, it has been reported that leucine regulates insulin secretion by modulating adrenergic α_2_ receptors through the mTOR pathway (Yang et al., 2012). This finding highlights the system's ability to direct the balance between leucine‐KIC toward different targets. This balance seems to be of high physiological relevance insofar as the effects of KIC are in the short‐term range (K‐ATP inhibition), while those of leucine imply that mTOR affects long‐term processes such cell growth and proliferation. For this study, it is worth noting that the KIC‐leucine data point to the involvement of the distal components of the canonical insulin secretion pathway centered on the consequences of the K‐ATP channel modulation. It is also worth noting that previous considerations about the role of the leucine‐KIC pair have supported the hypothesis based on a functionally regulated balance between KIC transamination to leucine and its catabolism to increase cytosolic Ca^2+^ and insulin secretion (Gao et al., 2003). Our results appear to be more consistent with the hypothesis of functional heterogeneity of pancreatic β‐cells in their response to leucine (Hiriart et al., 1995).

Moreover, we tested the effect of palmitate, a fatty acid that exhibits a particular behavior: the insulin secretion induced by palmitate depends on glucose concentration (Carpinelli, Picinato, Stevanato, Oliveira, & Curi, 2002). At low glucose concentrations (5.6 mM), palmitate reduces insulin secretion while promoting it at high levels (16.7 mM). The reason for this effect is that at low glucose concentrations, palmitate diverts glycerol‐phosphate into lipid synthesis, which reduces glucose from the glycolytic flux, and then reduces glucose oxidation and ATP production. At high glucose concentrations, the glycolytic flux is sufficient to provide glycerol‐phosphate for lipid synthesis and carbons for the Krebs cycle. The experiments with palmitate were performed at low glucose concentrations (8 mM), then, as expected, with palmitate, TPS did not occur.

Two well‐known pharmacological tools have been used to evaluate the distal step of glucose metabolization: diazoxide and tolbutamide. When carbachol was applied together with glucose and diazoxide, TPS was not induced. Since diazoxide does not prevent glucose intake or metabolization (Aizawa et al., 1994; Gembal, Gilon, & Henquin, 1992; Kharade et al., 2019), these data reveal the need for the requirement of membrane depolarization and calcium entry to induce TPS. Tolbutamide, an antidiabetic drug, acts in a manner opposite to diazoxide, blocking ATP‐K channels, depolarizing cells, and inducing the opening of calcium channels and insulin secretion even in the absence of glucose. Carbachol, in the presence of tolbutamide, did not require glucose to induce TPS, which supports the role of glucose to increase the intracellular calcium concentration; the other intermediate metabolic steps are unnecessary for this purpose.

It has been shown that kinases are critical for β‐cells to function and participate in cholinergic pathways to regulate insulin secretion (Persaud, Jones, & Howell, 1991) and generate sustained cellular responses in the islets of Langerhans (Alkon & Rasmussen, 1988; Rasmussen et al., 1995). The execution of the basic protocol (glucose and carbachol) in the presence of the nonspecific inhibitor of kinases staurosporine shows that the induction of TPS is prevented, suggesting the participation of kinases. Specifically, the action of PKC in pancreatic β‐cells is complex, inhibits phase 1 and stimulates phase 2 of insulin secretion induced by both glucose and carbachol (Thams, Capito, Hedeskov, & Kofod, 1990). To test the possible role of this kinase, we conducted experiments in the presence of the activator of PKC PMA. PMA was not able to induce potentiation. This suggests that staurosporine may inhibit other targets that play a role in this TPS process. More research should be conducted to clarify the role of PKC and other kinases in the induction of TPS.

**Figure 15:**
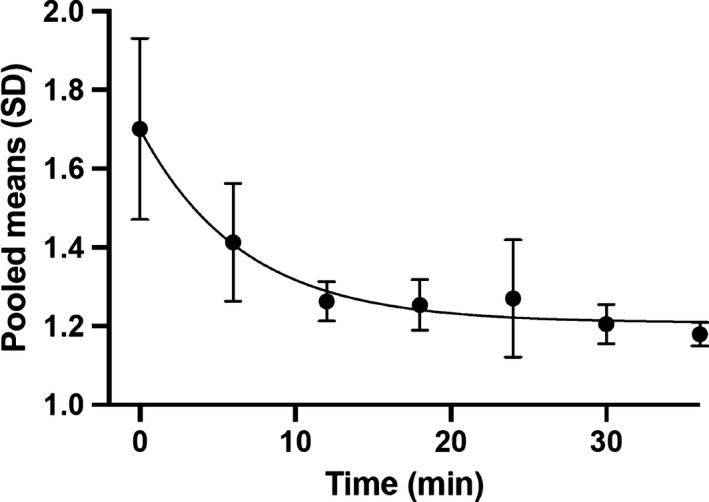
Fit to a single exponential decay of the AUD pooled means from the cases in which TPS was induced (basic carbachol protocol, glyceraldehyde, KIC, leucine, tolbutamide, cycloheximide; *n* = 32). Data show mean, *SD*. R squared goodness of fit of 0.98

The data presented also highlight distal mechanisms as candidates responsible for TPS induction. However, we cannot specify which one is responsible for the effect. In any event, it is interesting to observe the result of pooling the means of the AUDs of the different conditions where we have found TPS (basic carbachol protocol, glyceraldehyde, KIC, leucine, tolbutamide, cycloheximide) (cells, *n* = 32). As shown in Figure 15, one phase decay nonlinear fitting provides R squared goodness of fit of 0.98, which allows us to suggest that a single mechanism underlies at least the deactivation of TPS.

### 4.1 TPS as a case of cellular associative conditioning

One question arises from the data presented: can TPS be considered as associative cellular conditioning? Until now, the concept of memory has been restricted to neural systems and immunity. The TPS in β‐cells is limited under a behavioral scope, but meets the requirements to be considered Hebbian (Nicoll, Malenka, & Kauer, 1990), and shows parallels with other mechanisms of cellular memory, such as the induction of long‐term potentiation and associative learning in the Β‐photoreceptor of *Hermissenda crassicornis *(Alkon et al., 1992) or the abdominal ganglia of *Aplysia californica *(Kandel & Schwartz, 1982). In all these cases, properly paired stimuli can modify the response capacity of the system to one of them. Further, one of the inputs can be replaced by the calcium load induced by depolarization, and potentiation is impaired by membrane hyperpolarization even in the presence of properly paired stimulation. Our finding reveals a possible generalization of mechanisms of associative conditioning to non‐neural systems. In this case, the duration (up to one hour) and the lack of cycloheximide action, which indicates the absence of de novo proteins for the TPS induction, suggest that it is a short‐term process (Burgoyne, 1989; Schwartz & Greenberg, 1987).

### 4.2 Physiological implications

Although the main effect of muscarinic agents is to enhance glucose‐induced insulin secretion (Sanchez‐Andres et al., 1988), it seems that the integrated action of the parasympathetic system is much more complicated (Woods & Porte, 1978). Several observations support this possibility: (a) a given amount of glucose is more effective in stimulating secretion when given orally than when administered intravenously (Louis Sylvestre, 1976), which points to the involvement of neural mechanisms superimposed on the humoral; (b) atropine can inhibit the insulin response to ingested glucose, while not affecting the secretory response to infused glucose (Henderson, Jefferys, Jones, & Stanley, 1976); (c) vagotomy suppresses the development of diet‐induced obesity (Sclafani, Aravich, & Landman, 1981). The best known parasympathetically mediated physiological event is probably the reflex stimulation of insulin secretion (also called preabsortive or cephalic) that occurs before food enters the gut like a reflex salivary secretion. Classically, this increase in insulin secretion has been interpreted as being preparatory for the impending absorption of nutrients. To the extent that the experimental conditions for inducing TPS replicate those of the cephalic phase of insulin secretion (vagal discharge on basal glycemia), the physiological interpretation of this phase may be reconsidered. The cephalic phase of insulin secretion would play the classically assumed preparatory role, but it would also be responsible for establishing the sensitivity (gain) of the system to glucose. This gain would be at its highest point together with meals, returning the gain to baseline levels between meals when the physiological requirement must be in the opposite direction: maintaining a lower sensitivity, secreting insulin minimally, since the direction of the energy flux must be from the stores to the blood. This possibility adds complexity and enriches the physiological capabilities of cholinergic action, which has recently expanded with the observation that muscarinic agonist pulse trains and synchronizes the islets of Langerhans. As stated in other studies (Adablah, Vinson, Roper, & Bertram, 2019), this would result in coordinated insulin secretion of the islet population. Our data highlight a lasting coordination after a muscarinic pulse (or vagal discharge).

Furthermore, at the level of cellular physiology, our data suggest that the pancreatic β‐cell can integrate a neural signal to sustainably modify its responsiveness. This integration would endow these cells with the properties of coincidence detection (when the two inputs act in proper correlation), and prediction capability (the cells were ready for glucose challenges before glucose increased in the blood). The results of this research point to a generalization of the concept of associative conditioning being exhibited even in non‐neural tissues, which show particularities depending on the physiological demands to be subserved. In the case of β‐cell TPS, a short‐term memory process, increases the gain (sensitivity) of the system during meals, keeping it low between meals, and adapting the system to the cyclic nature of food intake.

## CONFLICT OF INTEREST

The authors declare that there is no conflict of interest.

## Author Contributions

Participated in research design: Sanchez‐Andres. Conducted experiments: Sanchez‐Andres, Pomares. Performed data analysis: Sanchez‐Andres, Pomares. Wrote or contributed to the writing of the manuscript: Sanchez‐Andres, Pomares, Malaisse.

## References

[phy214403-bib-0001] Adablah, J. E. , Vinson, R. , Roper, M. G. , & Bertram, R. (2019). Synchronization of pancreatic islets by periodic or non‐periodic muscarinic agonist pulse trains. PLoS ONE, 14(2), e0211832. 30726280 10.1371/journal.pone.0211832 PMC6364940

[phy214403-bib-0002] Aizawa, T. , Sato, Y. , Ishihara, F. , Taguchi, N. , Komatsu, M. , Suzuki, N. , … Yamada, T. (1994). ATP‐sensitive K^+^ channel‐independent glucose action in rat pancreatic β‐cell. American Journal of Physiology, 266(3 Pt 1), C622–C627. 8166224 10.1152/ajpcell.1994.266.3.C622

[phy214403-bib-0003] Alkon, D. L. , & Rasmussen, H. (1988). A spatial temporal model of cell activation. Science, 239(4843), 998–1005. 2830669 10.1126/science.2830669

[phy214403-bib-0004] Alkon, D. L. , Sanchez‐Andres, J. V. , Ito, E. , Oka, K. , Yoshioka, T. , & Collin, C. (1992). Long‐term transformation of an inhibitory into an excitatory GABAergic synaptic response. Proceedings of the National Academy of Sciences of the United States of America, 89(24), 11862–11866. 1334550 10.1073/pnas.89.24.11862 PMC50657

[phy214403-bib-0005] Andreu, E. , Soria, B. , & Sanchez‐Andres, J. V. (1997). Oscillation of gap junction electrical coupling in the mouse pancreatic islets of Langerhans. Journal of Physiology, 498(Pt 3), 753–761. 9051586 10.1113/jphysiol.1997.sp021899 PMC1159191

[phy214403-bib-0006] Ashcroft, F. M. , & Rorsman, P. (1989). Electrophysiology of the pancreatic β‐cell. Progress in Biophysics and Molecular Biology, 54(2), 87–143. 2484976 10.1016/0079-6107(89)90013-8

[phy214403-bib-0007] Atwater, I. , Carroll, P. , & Xu Li , M. (1989). Electrophysiology of the pancreatic β‐cell. In Molecular and Cellular Biology of Diabetes Mellitus, vol. 1. Ed. Draznin B, Melmed S, Leroith D, pp. 49–68. Alan R. Liss Inc, New York.

[phy214403-bib-0008] Bergman, R. N. , & Miller, R. E. (1973). Direct enhancement of insulin secretion by vagal stimulation of the isolated pancreas. American Journal of Physiology, 225(2), 481–486. 4722412 10.1152/ajplegacy.1973.225.2.481

[phy214403-bib-0009] Bliss, T. V. , & Collingridge, G. L. (1993). A synaptic model of memory: Long term potentiation in the hippocampus. Nature, 361(6407), 31–39. 8421494 10.1038/361031a0

[phy214403-bib-0010] Bliss, T. V. , & Lomo, T. (1973). Long‐lasting potentiation of synaptic transmission in the dentate area of the anesthetized rabbit following stimulation of the perforant path. Journal of Physiology, 232(2), 331–356. 4727084 10.1113/jphysiol.1973.sp010273 PMC1350458

[phy214403-bib-0011] Burgoyne, R. D. (1989). A role for membrane inserted protein kinase C in cellular memory? Trends in Biochemical Sciences, 14(3), 87–88. 2728104 10.1016/0968-0004(89)90126-6

[phy214403-bib-0012] Carpinelli, A. R. , Picinato, M. C. , Stevanato, E. , Oliveira, H. R. , & Curi, R. Insulin secretion induced by palmitate–a process fully dependent on glucose concentration. Diabetes Metab 28(6 Pt 2): 3S37–3S44, 2002. 12688632

[phy214403-bib-0013] Cerasi, E. (1981). Differential actions of glucose on insulin release: revaluation of a mathematical model. In: Carbohydrate Metabolism. Ed. Cobelli C and Bergman RN pp. 3. Wiley and Sons, New York.

[phy214403-bib-0014] de Lartigue, G. (2016). Role of the vagus nerve in the development and treatment of diet‐induced obesity. Journal of Physiology, 594(20), 5791–5815. 26959077 10.1113/JP271538 PMC5063945

[phy214403-bib-0015] Frohman, L. A. , Ezdinli, E. Z. , & Javid, R. (1967). Effect of vagotomy and vagal stimulation on insulin secretion. Diabetes, 16(7), 443–448. 5339250 10.2337/diab.16.7.443

[phy214403-bib-0016] Gagerman, E. , Idahl, L. Å. , Meissner, H. P. , & Täljedal, I. B. (1978). Insulin release, cGMP, cAMP, and membrane potential in acetylcholine‐stimulated islets. American Journal of Physiology, 235, E493–E500. 215036 10.1152/ajpendo.1978.235.5.E493

[phy214403-bib-0017] Gao, Z. , Young, R. A. , Li, G. , Najafi, H. , Buettger, C. , Sukumvanich, S. S. , … Matschinsky, F. M. (2003). Distinguishing features of leucine and alpha‐ketoisocaproate sensing in pancreatic beta‐cells. Endocrinology, 144(5), 1949–1957. 12697702 10.1210/en.2002-0072

[phy214403-bib-0018] Gembal, M. , Gilon, P. , & Henquin, J. C. (1992). Evidence that glucose can control insulin release independently from its action on ATP‐sensitive K^+^ channels in mouse β‐cells. J Clin Invest, 89(4), 1288–1295. 1556189 10.1172/JCI115714 PMC442990

[phy214403-bib-0019] Gilon, P. , & Henquin, J. C. (2001). Mechanisms and physiological significance of the cholinergic control of pancreatic β‐cell function. Endocrine Reviews, 22(5), 565–604. 11588141 10.1210/edrv.22.5.0440

[phy214403-bib-0020] Gilon, P. , Nenquin, M. , & Henquin, J. C. (1995). Muscarinic stimulation exerts both stimulatory and inhibitory effects on the concentration of cytoplasmic Ca^2+^ in the electrically excitable pancreatic β‐cell. The Biochemical Journal, 311(Pt 1), 259–267. 7575463 10.1042/bj3110259 PMC1136147

[phy214403-bib-0021] Heissig, H. , Urban, K. A. , Hastedt, K. , Zünkler, B. J. , & Panten, U. (2005). Mechanism of the insulin‐releasing action of alpha‐ketoisocaproate and related alpha‐keto acid anions. Molecular Pharmacology, 68(4), 1097–1105. 16014804 10.1124/mol.105.015388

[phy214403-bib-0022] Henderson, J. R. , Jefferys, D. B. , Jones, R. H. , & Stanley, D. (1976). The effect of atropine on the insulin release caused by oral and intravenous glucose in human subjects. Acta Endocrinol (Copenh), 83(4), 772–780. 1036650 10.1530/acta.0.0830772

[phy214403-bib-0023] Henquin, J. C. (2000). Triggering and amplifying pathways of regulation of insulin secretion by glucose. Diabetes, 49(11), 1751–1760. 11078440 10.2337/diabetes.49.11.1751

[phy214403-bib-0024] Hiriart, M. , Sanchez‐Soto, M. C. , Ramirez‐Medeles, M. C. , & Malaisse, W. J. (1995). Functional heterogeneity of single pancreatic beta‐cells stimulated by L‐leucine and the methyl ester of succinic or glutamic acid. Biochemical and Molecular Medicine, 54(2), 133–137. 8581358 10.1006/bmme.1995.1019

[phy214403-bib-0025] Kajinuma, H. , Kaneto, A. , Kuzuya, T. , & Nakao, K. (1968). Effects of methacholine on insulin secretion in man. Journal of Clinical Endocrinology and Metabolism, 28(9), 1384–1388. 4878435 10.1210/jcem-28-9-1384

[phy214403-bib-0026] Kandel, E. R. , & Schwartz, J. H. (1982). Molecular biology of learning: Modulation of transmitter release. Science, 218(4571), 433–443. 6289442 10.1126/science.6289442

[phy214403-bib-0027] Kaneto, A. , Kosaka, K. , & Nakao, K. (1967). Effects of stimulation of the vagus nerve on insulin secretion. Endocrinology, 80(3), 530–536. 6020219 10.1210/endo-80-3-530

[phy214403-bib-0028] Kharade, S. V. , Sanchez‐Andres, J. V. , Fulton, M. G. , Shelton, E. J. , Blobaum, A. L. , Engers, D. W. , … Denton, J. S. (2019). Structure‐activity relationships, pharmacokinetics, and pharmacodynamics of the Kir6.2/SUR1‐specific channel opener, VU0071063. Journal of Pharmacology and Experimental Therapeutics, 370(3), 350–359. 31201216 10.1124/jpet.119.257204 PMC6691189

[phy214403-bib-0029] Louis Sylvestre, J. (1976). Preabsorptive insulin release and hypoglycemia in rats. American Journal of Physiology, 230(1), 56–60. 1251910 10.1152/ajplegacy.1976.230.1.56

[phy214403-bib-0030] Malaisse, W. J. (1986). Stimulus‐secretion coupling in the pancreatic β‐cell: The cholinergic pathway for insulin release. Diabetes/Metabolism Reviews, 2(3–4), 243–259. 3017655 10.1002/dmr.5610020303

[phy214403-bib-0031] Malaisse, W. , Malaisse Lagae, F. , Wright, P. H. , & Ashmore, J. (1967). Effects of adrenergic and cholinergic agents upon insulin secretion in vitro. Endocrinology, 80(5), 975–978. 5337153 10.1210/endo-80-5-975

[phy214403-bib-0032] Nesher, R. , & Cerasi, E. (1987). Biphasic insulin release as the expression of combined inhibitory and potentiating effects of glucose. Endocrinology, 121(3), 1017–1024. 3304974 10.1210/endo-121-3-1017

[phy214403-bib-0033] Nesher, R. , Eylon, L. , Segal, N. , & Cerasi, E. (1989). Β‐cell memory to insulin secretagogues: Characterization of the time‐dependent inhibitory control system in the isolated rat pancreas. Endocrinology, 124(1), 142–148. 2462485 10.1210/endo-124-1-142

[phy214403-bib-0034] Nesher, R. , Praiss, M. , & Cerasi, E. (1988). Immediate and time‐dependent effects of glucose on insulin release: Differential calcium requirements. Acta Endocrinol (Copenh), 117(3), 409–416. 3289304 10.1530/acta.0.1170409

[phy214403-bib-0035] Newsholme, P. , Brennan, L. , & Bender, K. (2006). Amino acid metabolism, β‐cell function, and diabetes. Diabetes, 55(Supplement 2), S39–S47.

[phy214403-bib-0036] Nicoll, R. A. , Malenka, R. C. , & Kauer, J. A. (1990). Functional comparison of neurotransmitter receptor subtypes in mammalian central nervous system. Physiological Reviews, 70(2), 513–565. 1690904 10.1152/physrev.1990.70.2.513

[phy214403-bib-0037] Overduin, J. , Dworkin, B. R. , & Jansen, A. (1997). Introduction and commentary to: M.I. Mityushov (1954) "Conditioned reflex secretion of insulin". Integrative Physiological and Behavioral Science, 32(3), 228–246. 9322113 10.1007/BF02688621

[phy214403-bib-0038] Panten, U. , Kriegstein, E. , Poser, W. , Schönborn, J. , & Hasselblatt, A. (1972). Effects of L‐leucine and alpha‐ketoisocaproic acid upon insulin secretion and metabolism of isolated pancreatic islets. FEBS Letters, 20(2), 225–228. 11946423 10.1016/0014-5793(72)80801-9

[phy214403-bib-0039] Persaud, S. J. , Jones, P. M. , & Howell, S. L. (1991). Activation of protein kinase C is essential for sustained insulin secretion in response to cholinergic stimulation. Biochimica Et Biophysica Acta, 1091(1), 120–122. 1995062 10.1016/0167-4889(91)90231-l

[phy214403-bib-0040] Planelles, J. , & Luwisch, D. (1935). La acción hipoglucemiante del apetito, reflejo condicionado. Archivos De Neurobiología XVI, 383–386.

[phy214403-bib-0041] Planelles, J. , & Luwisch, D. (1936). Die blutzuckersenkende wirkung des appetits, ein bedingter reflex. Klinische Wochenschrift, 25, 1076–1077.

[phy214403-bib-0042] Porte, D. Jr , Girardier, L. , Seydoux, J. , Kanazawa, Y. , & Posternak, J. (1973). Neural regulation of insulin secretion in the dog. Journal of Clinical Investigation, 52(1), 210–214. 4682384 10.1172/JCI107168 PMC302245

[phy214403-bib-0043] Rasmussen, H. , Isales, C. M. , Calle, R. , Throckmorton, D. , Anderson, M. , Gasalla Herraiz, J. , & McCarthy, R. (1995). Diacylglycerol production, Ca^2+^ influx, and protein kinase C activation in sustained cellular responses. Endocrine Reviews, 16(5), 649–681. 8529575 10.1210/edrv-16-5-649

[phy214403-bib-0044] Rasmussen, H. , Zawalich, K. C. , Ganesan, S. , Calle, R. , & Zawalich, W. S. (1990). Physiology and pathophysiology of insulin secretion. Diabetes Care, 13(6), 655–666. 2192849 10.2337/diacare.13.6.655

[phy214403-bib-0045] Ribalet, B. , & Beigelman, P. M. (1981). Effects of divalent cations on β‐cell electrical activity. American Journal of Physiology, 241(1), C59–67. 7018263 10.1152/ajpcell.1981.241.1.C59

[phy214403-bib-0046] Sanchez‐Andres, J. V. , Gomis, A. , & Valdeolmillos, M. (1995). The electrical activity of mouse pancreatic β‐cells recorded in vivo shows glucose‐dependent oscillations. Journal of Physiology, 486(Pt 1), 223–228. 7562637 10.1113/jphysiol.1995.sp020804 PMC1156510

[phy214403-bib-0047] Sanchez‐Andres, J. V. , Malaisse, W. J. , & Kojima, I. (2019). Electrophysiology of the pancreatic islet β‐cell sweet taste receptor TIR3. Pflügers Archiv ‐ European Journal of Physiology, 471, 647–654. 30552496 10.1007/s00424-018-2237-6

[phy214403-bib-0048] Sanchez‐Andres, J. V. , Ripoll, C. , & Soria, B. (1988). Evidence that muscarinic potentiation of insulin release is initiated by an early transient calcium entry. FEBS Letters, 231(1), 143–147. 2452098 10.1016/0014-5793(88)80719-1

[phy214403-bib-0049] Schwartz, J. H. , & Greenberg, S. M. (1987). Molecular mechanisms for memory: Second messenger induced modifications of protein kinases in nerve cells. Annual Review of Neuroscience, 10, 459–476. 10.1146/annurev.ne.10.030187.002331 3551762

[phy214403-bib-0050] Sclafani, A. , Aravich, P. F. , & Landman, M. (1981). Vagotomy blocks hypothalamic hyperphagia in rats on a chow diet and sucrose solution, but not on a palatable mixed diet. Journal of Comparative and Physiological Psychology, 95(5), 720–734. 7031104 10.1037/h0077830

[phy214403-bib-0051] Sharp, R. , Culbert, S. , Cook, J. , Jennings, A. , & Burr, I. M. (1974). Cholinergic modification of glucose‐induced biphasic insulin release in vitro. Journal of Clinical Investigation, 53(3), 710–716. 4591035 10.1172/JCI107609 PMC333051

[phy214403-bib-0052] Taniguchi, S. , Okinaka, M. , Tanigawa, K. , & Miwa, I. (2000). Difference in mechanism between glyceraldehyde‐ and glucose‐induced insulin secretion from isolated rat pancreatic islets. Journal of Biochemistry, 127(2), 289–295. 10731696 10.1093/oxfordjournals.jbchem.a022606

[phy214403-bib-0053] Thams, P. , Capito, K. , Hedeskov, C. J. , & Kofod, H. (1990). Phorbol‐ester‐induced down‐regulation of protein kinase C in mouse pancreatic islets. Potentiation of phase 1 and inhibition of phase 2 of glucose‐induced insulin secretion. The Biochemical Journal, 265(3), 777–787. 2407236 10.1042/bj2650777 PMC1133701

[phy214403-bib-0054] Trajkovski, M. , & Wollheim, C. B. (2016). Physiology: Microbial signals to the brain control weight. Nature, 534(7606), 185–187. 27279209 10.1038/534185a

[phy214403-bib-0055] Wang, J. H. , & Feng, D. P. (1992). Postsynaptic protein kinase C essential to induction and maintenance of long‐term potentiation in the hippocampal CA1 region. Proceedings of the National Academy of Sciences of the United States of America, 89(7), 2576–2580. 1557361 10.1073/pnas.89.7.2576 PMC48704

[phy214403-bib-0056] Woods, S. C. (1972). Conditioned hypoglycemia: Effect of vagotomy and pharmacological blockade. American Journal of Physiology, 223(6), 1424–1427. 4641634 10.1152/ajplegacy.1972.223.6.1424

[phy214403-bib-0057] Woods, S. C. (1983). Conditioned hypoglycemia and conditioned insulin secretion. Advances in Metabolic Disorders, 10, 485–495. 6364723 10.1016/b978-0-12-027310-2.50026-8

[phy214403-bib-0058] Woods, S. C. (2013). From conditioned hypoglycemia to obesity: Following the data. Physiology & Behavior, 121, 19–24. 23352822 10.1016/j.physbeh.2013.01.010

[phy214403-bib-0059] Woods, S. C. , Alexander, K. R. , & Porte, D. Jr (1972). Conditioned insulin secretion and hypoglycemia following repeated injections of tolbutamide in rats. Endocrinology, 90(1), 227–231. 5009061 10.1210/endo-90-1-227

[phy214403-bib-0060] Woods, S. C. , Hutton, R. A. , & Makous, W. (1970). Conditioned insulin secretion in the albino rat. Proceedings of the Society for Experimental Biology and Medicine, 133(3), 964–968. 4907937 10.3181/00379727-133-34605

[phy214403-bib-0061] Woods, S. C. , & Porte, D. Jr (1974). Neural control of the endocrine pancreas. Physiological Reviews, 54(3), 596–619. 4601624 10.1152/physrev.1974.54.3.596

[phy214403-bib-0062] Woods, S. C. , & Porte, D. Jr (1978). The central nervous system, pancreatic hormones, feeding, and obesity. Adv Metab Disord, 9, 283–312. 347907 10.1016/b978-0-12-027309-6.50020-3

[phy214403-bib-0063] Wu, J. X. , Ding, D. , Wang, M. , & Chen, L. (2020). Structural insights into the inhibitory mechanism of insulin secretagogues on the pancreatic ATP‐sensitive potassium channel. Biochemistry, 59, 18–25. 31566370 10.1021/acs.biochem.9b00692

[phy214403-bib-0064] Yang, J. , Dolinger, M. , Ritaccio, G. , Mazurkiewicz, J. , Conti, D. , Zhu, X. , & Huang, Y. (2012). Leucine stimulates insulin secretion via down‐regulation of surface expression of adrenergic α2A receptor through the mTOR (mammalian target of rapamycin) pathway: Implication in new‐onset diabetes in renal transplantation. Journal of Biological Chemistry, 287(29), 24795–24806. 22645144 10.1074/jbc.M112.344259 PMC3397906

[phy214403-bib-0065] Zawalich, W. S. , Diaz, V. A. , & Zawalich, K. C. (1988). Role of phosphoinositide metabolism in induction of memory in isolated perifused rat islets. American Journal of Physiology, 254(5 Pt 1), E609–E616. 2834960 10.1152/ajpendo.1988.254.5.E609

[phy214403-bib-0066] Zawalich, W. S. , & Zawalich, K. C. (1988). Induction of memory in rat pancreatic islets by tolbutamide. Dependence on ambient glucose level, calcium, and phosphoinositide hydrolysis. Diabetes, 37(6), 816–823. 2838355 10.2337/diab.37.6.816

[phy214403-bib-0067] Zawalich, W. S. , Zawalich, K. C. , & Rasmussen, H. (1989). Cholinergic agonists prime the β‐cell to glucose stimulation. Endocrinology, 125(5), 2400–2406. 2676484 10.1210/endo-125-5-2400

